# Exploring the influence of socio-cultural factors and environmental resources on the health related quality of life of children and adolescents after congenital heart disease surgery: parental perspectives from a low middle income country

**DOI:** 10.1186/s41687-020-00239-0

**Published:** 2020-08-28

**Authors:** Laila A. Ladak, Robyn Gallagher, Babar S. Hasan, Khadija Awais, Ahmed Abdullah, Janice Gullick

**Affiliations:** 1 Department of Paediatrics and Child Health, School of Nursing and Midwifery, The Aga Khan University, Karachi, Pakistan; 2 Susan Wakil School of Nursing and Midwifery, Sydney Nursing School, The University of Sydney, Sydney, Faculty of Medicine and Health, Sydney, New South Wales Australia; 3 Charles Perkins Centre, Sydney Nursing School Faculty of Medicine and Health, Sydney, New South Wales Australia; 4 Department of Paediatrics and Child Health, The Aga Khan University, Karachi, Pakistan; 5 Medical College, The Aga Khan University, Karachi, Pakistan

**Keywords:** Health related quality of life, Congenital heart disease, Surgery, Parental perceptions, Environment, Sociocultural factors, Low middle income countries

## Abstract

**Background:**

Health related quality of life (HRQOL) is an important indicator of long-term well-being, influenced by environmental factors such as family, culture, societal norms and available resources. This study aimed to explore parental perspectives on the influence of socio-cultural factors and environmental resources on the HRQOL of children and adolescents after congenital heart disease (CHD) surgery.

**Methods:**

Using a descriptive, qualitative design, semi-structured interviews of children/adolescents who had CHD surgery in this low-middle income country (LMIC) were collected between July to December 2017. There were 20 families enrolled, which included 18 parent dyads (mother and father) and two single mothers, making a total of 38 participants. Initial inductive analysis was further refined using the Social Ecological Model as an analytic lens.

**Results:**

At the *intrapersonal level*, unrealistic expectations of surgery, residual CHD symptoms and difficulty maintaining educational progress were of great concern. There were low levels of health literacy and understanding about CHD among family and friends, however, strong kinship ties were an important resource at the *interpersonal level*. These families lived in poverty and mothers often carried the sole burden of care for their sick children. At the *institutional level*, there were unclear expectations of the child’s needs at school, and parents had poor access to psychological, family-planning and genetic counselling, and poor access to CHD education resources. At a *sociocultural level*, religion and trust in God were important coping factors, however, CHD was a gendered experience with particular concerns around scarring and the marriageability of girls. Parents noted the deficit of antenatal and specialist CHD services and felt the consequence of a lack of a universal health care system at the *public policy level*.

**Conclusion:**

Socio-ecological factors have the potential to explain the issues and challenges that children living in LMIC experience with CHD after surgery. The study findings will help to inform future interventions to be implemented in countries like Pakistan.

## Introduction

Better and earlier diagnostics and clinical management of congenital heart disease (CHD) have led to decreased mortality and increased survival [1]. However, CHD patients frequently need life-long care, making optimal health related quality of life (HRQOL) an important goal [2]. Reduced HRQOL in children and adolescents with CHD is well-documented in high-income countries (HICs) [3, 4], however, data is lacking from low middle income countries (LMICs) [5]. Differences in the nature and availability of resources challenge the notion that findings from HICs are directly applicable to LMICs. Evidence for these differences are indicated in the only report from a LMIC [6] which described much lower emotional, physical and psychosocial functioning in a Pakistani cohort than that reported in a meta-analysis from HICs. In that study, 129 CHD surgical patients from Pakistan were compared with their age-matched siblings and found that compared to HIC, differences in HRQOL encompassed emotional and physical functioning, and worse cardiopulmonary symptoms like breathlessness and tachycardia. This paper reports qualitative findings from the larger program of mixed methods research of HRQOL in CHD surgical patients in Pakistan. Quantitative findings are reported elsewhere [6]. This qualitative work seeks to provide social, resource-oriented and health system contexts for these published quantitative findings.

Pakistan is a LMIC with a population of over 215 million, and an estimated 50,000 live births complicated by CHD annually. However, CHD incidence may be underestimated due to a higher proportion of home birth rates leading to limited early health professional contact [7]. Sociocultural and resource influences are thought to contribute to higher CHD rates in Pakistan. Apart from economic influences, Pakistan is a patrilineal society where marriage to a paternal uncle’s child is frequently sought. While this intensifies family ties, it is thought to double the risk of congenital birth defects.

Despite the huge CHD burden in Pakistan, only 5% of the CHD population receive the standard of health care recommended in practice guidelines from HICs [8]. This is primarily due to a relatively poor supply of expert staff and health services. For example, there are only six hospitals providing paediatric cardiac care, eight paediatric cardiac surgeons and 25 paediatric cardiologists in Pakistan [8]. In contrast, HICs are better resourced, with the USA providing a paediatric cardiac care facility for every 120,000 people [9].

A diverse array of resources are associated with better HRQOL in HICs, including more family support, higher income and education levels as well as higher gross domestic product and better health care systems [3, 4]. Assessing the perceptions of parents may help to further understand the context of CHD surgical patients’ HRQOL outcomes in a resource-constrained environment.

Parents’ perceptions about their child’s HRQOL following CHD surgery has many subcomponents and contributing factors can be complex and interactive. The World Health Organization (WHO) reinforces the impact of the conditions in which people are born and grow, their surrounding community and culture and the distribution of resources on their health and HRQOL [10]. For this reason, a theoretical lens that helps focus on these environmental concerns is useful to systematise research analysis. The Social Ecological Model [11] has therefore been used here to explore the impact of interventions on parental experiences and perceptions.

We were interested in the perceptions of parents to help us understand complex CHD pre-surgical care, decision-making about surgery, and the care after hospital discharge. Given parents’ key role in the lives of children, we hoped we might also understand, by proxy, some of the differences in children’s HRQOL findings for this LMIC cohort. The aim of this paper is, therefore, to draw on the five levels of analysis in the Social Ecological Model to further understand the influence of individual, sociocultural and environmental factors that influence health-related decision-making and HRQOL for children or adolescents after CHD surgery in Pakistan.

## Methods

A descriptive, qualitative design was used to explore the parental perspectives of the influence of socio-cultural factors and environmental resources on the HRQOL of children and adolescents after CHD surgery.

### The theoretical framework

**Fig. 1:**
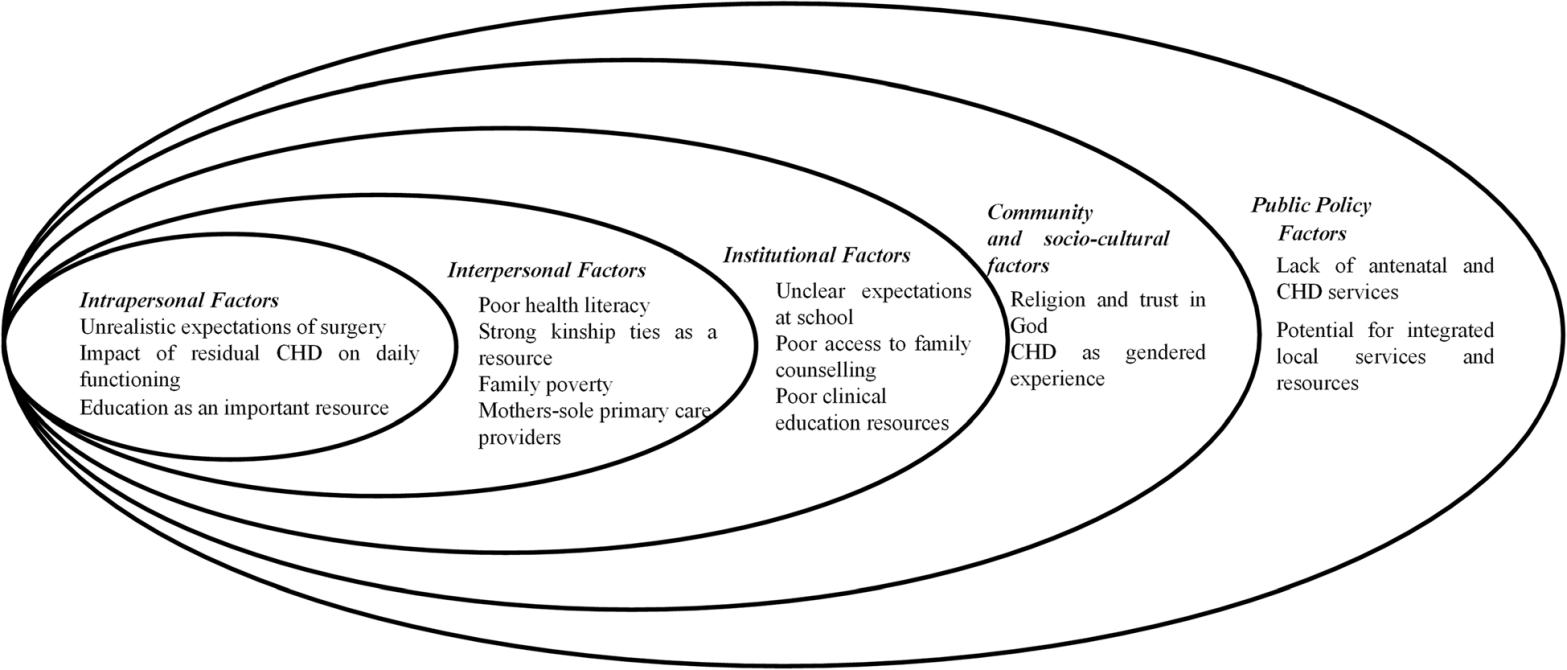
Sociological model-Parental perspectives on the socio-cultural and environmental factors influencing the HRQOL of CHD surgical patients

The Social Ecological Model (SEM) [11] was initially developed as a framework for understanding health promotion activities. The SEM has since been used to explore people’s responses to a range of health interventions. In this paper, it is used to understand and describe the context for experiences and HRQOL after CHD surgery in Pakistan. This assumes that the following five domains of analysis create the context and capacities for health experiences and health improvements strategies: *1) intrapersonal factors* (this may include personal demographics, skills and attitudes - in this case of either parent or child - and the knowledge about and expected value of an intervention), *2) interpersonal processes* and primary groups (this includes how social relationships with family, friends, work colleagues and acquaintances influence stress and coping, social roles and provide practical assistance), *3) institutional factors* (how organisations support behaviours that promote health and well-being, and the rules under which they operate), *4) community factors* (relationships between organisations and informal networks), and *5) public policy* (national policies/laws, national health resources and national health services) (See Fig. 1).

The benefit of using the SEM framework is that it tailors a range of ecological theories to public health structures. By identifying a range of factors that impact on and maintain health behaviours, it provides multiple points for intervention, while making visible the complexities and the subsequent requirement for sophisticated and multifactorial solutions to health delivery [12].

### Setting and participants

The study was conducted at a private, tertiary care hospital in Karachi, Pakistan, which caters for patients of diverse ethnic and socio-demographic backgrounds from across the country. The hospital provides financial support for medical and surgical management of families in need. Participants were parents of CHD patients aged 2–18 years who were able to understand English or Urdu and were willing to travel to the study setting for a face-to-face interview. This nested sample, a subset of parents from the larger study, were recruited using a stratified, purposive strategy to achieve diversity in CHD diagnoses (simple, moderate and complex CHD) and child age (children and adolescents). Parents from 27 families (both mother and father where possible) were approached. Seven pairs of parents declined to participate due to lack of time. Of the remaining families, data saturation was achieved with the 18th interview. Two additional interviews were completed, increasing participation to 20 families, but there were no further novel findings in these subsequent interviews. The 20 families included 18 parent dyads (mother and father) and two single mothers, to reach a total of 38 participants (see Table 1).

**Table 1:** Participants characteristics

	n	%
Dyads (both parents interviewed)	18	90
One parent interviewed (both were mothers)	2	10
**Mean age (years)**		
Father	43.27	
Mother	36.21	
**Education – Father (total 18)**		
Grade 10/12	10	55
Completed University degree	8	45
**Education – Mother (total 20)**		
Grade 10/12	16	80
Completed University degree	4	20
**Occupation – Father**		
Business	9	45
Service	9	45
Not alive / separated	2	10
**Occupation – Mother**		
Housewife	20	100
**Income:** Below poverty line (LMIC)	15	75
Above poverty line (LMIC)	5	25
**Family Structure**		
Nuclear	11	55
Extended	9	45
*Median number of siblings	3 (range 2–6)	
*Median number of co-dwelling family members	7 (range 4–16)	

**Note:** *Inclusive of patients

### Ethical considerations

Ethics approval was obtained from the study hospital ethics committee (3737-Ped-ERC-15). A participant information statement explaining the study purpose and procedures was available in both English and Urdu**.** The hospital’s paediatric department reimbursed participants’ travel costs. Informed consent was obtained from parents in writing. A participant distress protocol was available to guide researcher actions should a participant become distressed, but this was not required during the data collection. There was no pre-existing relationship between the interviewer and participants. All names are pseudonyms.

### Data collection

Data were collected between July to December, 2017. A semi-structured interview guide was developed based on a review of the literature and the first author’s (LL) clinical and cultural experience in the research setting. The interview guide has questions specific to the study aims (supplementary file 1). Face-to-face interviews were conducted in a private room of the study setting to facilitate free expression and to maintain confidentiality. There was one interview conducted per pair of participating parents, with a mean interview time of 50 min (range 30–60 min). Both the parents complemented each other’s experiences of parenting the CHD child and were comfortable sharing their issues and concerns. All interviews were conducted in Urdu (the local and most commonly spoken language in Pakistan) and were audio-recorded, transcribed in Urdu and translated to English (LL). Back translation of key exemplars was then used to refine and validate translation accuracy (BH). Clinical data (CHD diagnosis, severity, age at surgery, surgical procedures and New York Heart Association (NYHA) class) was retrieved from the hospital data management system, and socio-demographic data (parental age, education, income, family structure, urban versus rural residence) was collected from participants directly (Table 2).

**Table 2:** Characteristics of the CHD patients

	n	%
Children (2–9 years)	13	65
Adolescents (13–16 years)	7	35
Gender		
Male	12	60
Female	8	40
CHD complexity		
Simple	2	10
Moderate	7	35
Complex	11	55
One surgical procedure performed	13	65
≥ 2 surgical procedures performed	7	35
Median age at surgery (range) – years	1 (0–13)	
Median F up years following surgery - years	3.42 (1–7.5)	

### Data analysis

Qualitative content analysis techniques were applied [13, 14], with the Social Ecological Model (SEM) used as an analytic lens to guide analysis of parent narratives under individual, interpersonal, institutional, community and public policy domains. The ‘unit of analysis’ is the translated, transcribed parent interview text relating to elements of the social, familial, cultural, geographic and economic environment. The ‘context’ is the HRQOL of children and adolescents following surgery for CHD in Pakistan. Analysis was both inductive (where meaning emerges from the data) and deductive (where analysis is driven by the theoretical model). In the inductive phase, ‘meaning units’- segments of verbatim interview data containing a cohesive idea, were grouped according to similarities in content. These ‘meaning units’ were then condensed into shorter descriptions that remained ‘close to the text’, before applying an interpretation of the underlying meaning. These underlying meanings were then grouped so that inductively-derived subthemes [14] were deductively refined and organised using the theoretical domains of the SEM (See Table 3). Key themes were well saturated, with the data having the qualities of both *richness* (i.e. detailed nuanced and layered) and *thickness* (regarding the amount of data available to support each theme) [15]. To ensure themes were well-rounded, where available, we included negative case examples where a participant’s experience of a topic seems to contradict emerging patterns in the data to make visible the diversity of experiences and strengthen the description of a typical case [16]. Demographic data were analysed using descriptive statistics.

**Table 3:** Coding process framework using the domains of the Social Ecological Model [11]

Themes guided by SEM	Sub Themes	Categories	Examples of Quotations
***Intrapersonal Factors***	Expectations from CHD surgery	‣Unrealistic expectations of surgery	*“I thought after surgery … I wouldn’t have to worry about his heart problem and health anymore”*
		‣Impact of residual CHD on daily functioning	*“He is not able to play … gets angry …*”
		‣Education as an important individual strategic resource	*“He should get education from a good institute and achieve a good position in life”*
***Interpersonal Factors***	Health literacy and resource issues	‣Poor health literacy within social networks	“*Where did she get this disease from, what was the reason? Was it by birth or has it occurred now*?”
		‣Strong kinship ties as a resource	*“They [extended family] are concerned that my child should experience a good healthy life … The first thing they ask about is my child’s health”*
		‣Family poverty	*“I blame myself … My husband also feels so guilty that we are not able to fulfil her needs because of financial constraints”*.
		‣Mothers as sole primary care providers	*“The mother and the suffering child are left alone to fight and face the problems …*”
Institutional Factors	Lack of parental and teachers’ counselling	‣Unclear expectations at school	*“Teachers should be educated about the activities they [child with CHD] can perform … Or if any emergency situation arises, what they should do”*
		‣Poor access to family counselling	*“Parents should be told … not to plan for the next baby … If these things had been communicated … we would not have planned our baby”*
		‣Poor clinical educational resources	*“I wish these [post-surgery long term health issues] were communicated … We would have been mentally prepared for it.”*
Community and social–cultural factors	Religion and gender issues	‣Religion and trust in God	*“Allah has given this disease, so He is the one who will cure it …*”
		‣CHD as a gendered experience	“*… they [people] said that since she is a girl, there is no need for her to go through the surgery... and spend the huge cost”*
***Public Policy Factors***	Lack of CHD facilities across the country	‣Lack of antenatal and CHD services	*“There are very few hospitals for children with heart problems [in the country …*”
		‣Potential for integrated local services and resources	“*… workshops, training programs or activities arranged for children as well as parents.”*

#### Rigour

A content analysis checklist [13] was used to improve trustworthiness and to guide the preparation, organisation and reporting of the data with additional guidance from the COREQ (COnsolidated criteria for REporting Qualitative research) [17] (Supplementary file 2). Credibility is supported by maximum variation sampling for age, gender and CHD type and inclusion of verbatim exemplars. Dependability was enhanced by interviews collected within a confined period to reduce design-induced changes. Conformability was supported by initial independent coding by two researchers (LL, JG), later rechecking by another researcher (RG) and by the back translation of key exemplars to confirm accuracy (BH). Transferability is facilitated by a clear description of participant characteristics and a deliberate focus and description of this LMIC setting.

## Results

Table 3 displays the themes, sub-themes and categories developed under the SEM domains (Table 3). Most children and adolescents (*n* = 18, 90%) had moderate to complex CHD severity [18] with diagnosis occurring at a median age of 1-year (range 0–7 years), surgery at 0–11 years and a median follow-up time of 4.42 years. Sixty-five percent of the children and adolescents had a New York Heart Association (NYHA) class I, and 25% reported class II. More than half the children were taking cardiac medications (Supplementary file 3).

### Intrapersonal factors

#### Unrealistic expectations of surgery

Parent participants frequently described their initially unrealistic expectations of corrective surgery as a cure for complex defects. Consequently, parents were surprised when the child’s functional challenges continued, sometimes worsening and further reducing perceived HRQOL. The mother of Wahab (6-year-old male with Tetralogy of Fallot -TOF repair and multiple hospitalisations) explained: *“I thought he would be alright … But after this surgery, I had to face even more problems for him.”*

#### Impact of residual CHD on daily functioning

Parents described persistent, disabling CHD symptoms that had a pervasive effect on the daily functioning of their child/adolescent. For instance, altered sleeping habits due to tachycardia and tachypnoea had a perceived negative impact on growth, schooling and social inclusion and important religious milestones. The mother of Kashif (5-year-old boy with Blalock Taussig – BT shunt) explained the impact of fatigue on his learning: “*He gets exhausted … then sits quietly in a corner and doesn’t talk to anyone*.”

#### Education as an important individual strategic resource

Despite many participants living in poverty, 20% of the mothers and 45% of the fathers had a university degree. Educational achievement was seen as an important strategy to gain resources, maximise future earning potential, and bolster financial security. Therefore, when asked what they believed was important to their child’s HRQOL, parents of children and adolescents requiring surgery for CHD commonly identified current or future educational attainment as an important element. Parents then, logically, expressed great concern when schooling was interrupted by symptoms or surgery. The mother of 6-year-old Wahab exclaimed: *“How will he catch up with his peers in school and … cope with his studies? Learning to write, read and keeping pace in school is our utmost concern.”*

### Interpersonal factors

#### Poor health literacy within social networks

Participant’s narratives revealed often low levels of health literacy both generally and specifically for CHD in themselves and their family and friends. This poor understanding of the causes of CHD created a stigma that impacted children at an interpersonal level because people within the immediate social network reacted to the child in a way that classified the child at best as different, and at worst, dangerous.

As expressed by the father of Sajda (14 years female with TOF repair): “*They [neighbours and family members] say “Keep your child away from ours. I am afraid she will transmit this disease to my daughter*.”

#### Strong kinship ties as a resource

Approximately half the families lived in an extended family structure. Participants emphasised the significance of their strong kinship ties in providing emotional and moral support mitigating, at least in part, the stressors of parenting a child following CHD surgery. “*I am so thankful to God as my family members … supported me a lot.”*

#### Family poverty

Approximately two-thirds of these families were living below the poverty line [19]. Families discussed the considerable financial burden that arose from their child’s surgery and this added to their stress and uncertainty about their child’s care. The father of Nabeel (7-year-old boy with BT shunt) explained: *“My child’s operation cost 10 to 12 lacs rupees* [6,000 to 7,000 USD] *which I could not afford … On the one hand, I was worried about the disease and surgery and on the other hand about finances.”*

For some parents, only the initial surgical costs could be covered. This meant that any subsequent and necessary re-operation was not possible, leaving the affected child at risk of deterioration and parents feeling guilty. Money spent on CHD and surgery impacted the whole family resulting in restrictions in other areas of family expenditure. This could be a source of marital discord as the mother of Mehmood (15-year-old boy with Ventricular Septal Defect -VSD closure/Patent Ductus Arteriosus -PDA ligation) articulated: *“If a child has health issues … the money earned goes to hospital bills … fees and treatment, and there isn’t enough money left for life’s other necessities. As husband and wife, we started having frequent … fights because of this.”*

#### Mothers as sole primary care providers

It seemed that family and society had high expectations for the all-encompassing nature of the mothering role, made more difficult by the care needs of the sick child, often in a family with several siblings. This meant mothers experienced multiple caring stressors and were frequently isolated by their role as the sole primary care provider. This was most evident after the child was discharged from surgery and social isolation, fatigue and guilt were pronounced. Mehmood’s mother explained:

*“This is a big issue in our society, that a child is the complete responsibility of the mother … You cannot go anywhere. Your sleep pattern, social activities, everything gets disturbed … If you are giving* [the sick child] *special attention, then the other children* [siblings] *feel negative.”*

### Institutional factors

#### Unclear expectations at school

Poor understanding of the child with CHD’s capacity to participate in physical activity meant frequent overprotection at school, and activity restrictions, with the potential for pervasive negative effects on the child, socially and developmentally. The father of Irfan (6-year-old male with TOF repair) explained:

*“The sports teacher asked him to sit in one place during his sports session … If he feels neglected in school, he will lose interest which will create a very negative impact on his personality.”*

#### Poor access to family counselling

Psychological and other specialist counselling support was lacking, including counselling for family planning decisions. The lack of family planning support was of particular concern for parents with additional, young children, as the care of their sick child exacerbated the stress of their overall caring roles. Parents saw this as an important omission, given the sick child’s difficult journey, the heavy parenting demands, and the fact that that some congenital defects are hereditary. The father of Mehmood suggested: *“Parents should be counselled during pregnancy … Other than the cardiologist, a child should also have a psychologist consultation … Genetic counselling should be provided … if they are planning for another pregnancy.”*

#### Poor clinical education resources

The participants’ own poor understanding of CHD surgery suggested a lack of appropriate, formal information resources. They believed more information about the likely outcomes of surgery, delivered in suitable language, would help them prepare for their child’s outcome and care. Laila’ mother explained: *“There should be a brochure for the parents, with practical information about important things for the child’s health and family functioning after surgery.”*

### Community and socio-cultural factors

#### Religion and trust in God

Religion played a significant role in creating a sense of fatalism about CHD that shaped the illness and caring experience. Parents perceived CHD as the will of God, and therefore expressed their submission to God, considering His divine power. The mother of Fatima (9-year-old girl with BT shunt) explained: *“Life and death is in Allah’s control. All we can do is to provide the best to our child … a good quality of life.”*

In making decisions about surgery, three participants pursued “Istakhara” - an Islamic ritual for decision-making when a person is faced with a dilemma. They believe that through this act, the person receives an indication of God’s wish either through a resolution of obstacles, through obvious assistance from nature, and/or perceiving an answer through one’s dreams. As expressed by the father of Sajida (14-year-old female with complex CHD): *“We went for Istakhara which took us a few months and we got the indication to go for surgery”.*

While some participants considered their child’s CHD as their fate or God’s will, their faith in God also provided a sense of validation which, in turn, helped them cope with CHD. There was a sense that God would not let people suffer unnecessarily and that prayers and religious rituals could influence the survival and health of their child following surgery. People’s strong Islamic belief in God as the provider of everything was expressed by Laila’s mother: *“I always pray to God* [Allah] *… You are the biggest doctor of all... Everything is possible for You.”*

Indeed, for some participants, CHD was perceived in a positive way, sometimes as a divine test that ultimately strengthened people who faced such a trial. The mother of Perveen (15-year-old girl with VSD closure) explained:

*“We believe [Perveen] is special because God chooses those who can bear such a problem … [At first] I cried a lot … Then I saw children with worse health and thanked God that He has given us this hospital and she received treatment.”*

As a result of the impact of CHD on their child’s education, some parents replaced secular education with a common alternative path of religious scholarship. However, the mother of Abdullah (6-year-old boy with Glenn shunt) felt this option was no longer open to her son due to the cognitive challenges he was facing: *“I wanted to make him Hafiz e Quran* [a person who has memorized The Quran]*, but now I think that it would be very challenging for him* [cognitively]*. A normal child can do it.”*

#### CHD as a gendered experience

The influence of an overall societal view about the value and role of females was evident throughout the interviews, particularly in relation to marriage. For females, future marriage was described as a source of financial and social security, with accountability for a woman’s welfare eventually shifting from her parents to her husband and in-laws. If CHD and/or surgery became an obstacle to marriage, a female child could become destitute. The father of Kainat (13-year-old girl with TOF repair) explained his fears:

*“Who will take care of her when we are no longer in this world? We have heard people with heart issues do not … or should not get married. So this makes us worried that she is a girl. Who is going to look after her, and to what extent? These thoughts make us feel helpless.”*

Given the expected financial burden of CHD surgery, parents of females were faced with the opinions of family and friends as to whether the investment in surgery for girls would be worth the return. Family’s reservations about either the initial surgery or re-operation also arose from the non-curative nature of some surgery, and the potential increase in life-long dependency (versus earlier death). The mother of Lubna (7-year-old girl with TOF repair) explained: *“They* [family] *used to say now she will be dependent, and I will have to carry her in my arms for her whole life”.*

The value of a female who does marry is often measured by the work contribution she can make to her husband and his family. As a result, these parent participants feared their daughters with CHD would not be valued. Kainat’s mother explained: *“She will have more surgery and her hand is not working because of a stroke. So we are worried if she gets married, how she will manage with her in-laws and fulfil their expectations?”*

Not all participants faced this. The mother of Sumera (15-year-old girl with Fontan procedure) acknowledged the support and encouragement of her husband during the crucial period of their daughter’s diagnosis and surgery:

*“I am so thankful … especially to my husband … We didn’t think as our society thinks … we did what we should as parents. We went through two operations for our daughter without considering her status as a girl*.”

Because a girl’s appearance was vital to marriage prospects, the surgical scar was a pivotal factor in acceptance of the need for surgery. Some participants sensed their community prioritised avoidance of potential scarring above the child’s health needs and this was distressing. Sumera’s mother revealed: *“They believe … she shouldn’t have had surgery. ‘She is a girl and it looks so bad’ … They don’t realize the problem* [serious CHD] *behind this surgical scar …*”.

Mothers, therefore, assisted their daughters to hide their scar with either high neck clothes or a “hijab” (a scarf worn by Muslim females which covers the head, neck, shoulders and chest). According to the mother of 15-year-old Perveen: *“Girls wear open neck dresses, but she avoids it … and often complains she cannot wear good dresses …*”.

In striking contrast to the negative attitudes towards girls with CHD, parents reported their family and friends exhibiting a particular preciousness towards CHD-affected boys. Salman (2-year-old boy with Coarctation Of Aorta-COA correction) was the only male child in the family and his father expressed the influence of gender on the level of family support*: “He was born after my three daughters, that’s why no-one said anything to him … and had sympathy towards him.”*

Male circumcision is considered a significant socio-cultural ritual in Pakistan, normally performed on male children during the first days after birth. However, early diagnosis of CHD and the need for surgery could challenge expectations of parents and family around this normal cultural ritual. Father of Tanveer (3-year-old boy with Total Anomalous Pulmonary Venous Return - TAPVR) explained: *“My child did not have circumcision [musalmani] as doctors told us [to delay it] … All my family members ask me why it hasn’t been done.”*

### Public policy factors

#### Lack of antenatal and CHD services

Participants described poor availability of, and access to, specialised pediatric cardiac and antenatal health services in Pakistan, which had a substantial impact, particularly for rural patients and families. Diagnosis of CHD seemed to occur at a later age than normally reported in HIC settings. Qasim (7-year-old boy with Glenn shun, and complex CHD diagnosed at one week of age) lived in rural Pakistan. His father believed that had Qasim received his CHD diagnosis during the antenatal phase, the family would have been better prepared to face the emotional consequences, and more importantly, to manage the longer-term logistics of their child’s health:

“*If our child was diagnosed during pregnancy, or at birth, we would have been mentally ready and things could have been managed better … We have to take our child to Karachi [Pakistan’s main city] for his check-ups … If a problem occurs during the night, how we can rush to Karachi? … It takes 2-3 hours to get there.”*

#### Potential for integrated local services and resources

The lack of a publicly-funded health system contributed to family concerns about ongoing support for their child’s health problems. Several participants suggested potential solutions to improve aspects of HRQOL for their children, and their own parenting experience within this resource-constrained setting. These included follow-up phone calls from the hospital and the creation of support groups and community resources for patients and parents at the local level. The father of 6-year-old Irfan suggested structured learning activities for parents and children: *“We all could learn from each other … and would benefit from the experience of other parents.”*

## Discussion

To our knowledge, this is the first qualitative study exploring the influence of socio-cultural factors and environmental resources following paediatric CHD surgery in a LMIC. Among the *intrapersonal factors*, many parents reported the impact of residual CHD symptoms on their child’s engagement and success with learning activities. Education was identified as an important strategic resource that could elevate future possibilities for these children. Supporting educational attainment for children with CHD may reduce socially-embedded disadvantage by reducing future poverty, promoting economic independence and social respect and may empower women to overcome gender discrimination [20].

Education is also known to increase the age at which women marry, may heighten their control over household resources and increase their engagement in family planning important for the reduction of genetically inherited disease. In addition, the United Nations Sustainable Development Goals have highlighted quality education, good health, gender equality and poverty eradication as indicators of better HRQOL [21]. The importance of education in this study may also be influenced by the generally high educational attainment in this sample. There was variability in the ages of the CHD children in our study. The child’s age may influence parents perceptions about their HRQOL, and parents of younger children may be more protective concerning their child’s activities compared to parents of adolescents [22].

Among the important *interpersonal factors*, many participants lived in extended family structures and strong kinship ties were vital contributors to decision-making about health. This is congruent with research findings on antenatal screening attitudes in Pakistan: participants thought in-laws should be included in conversations about antenatal screening, even if ultimately the decision was the couple’s [23].

The family’s financial constraints seemed the major influence on decisions about, and attitudes towards, CHD surgery. This is contrary to HICs where parental decision-making towards CHD surgery are mostly influenced by information provision that empowered parents [24]. Though CHD cost burden is reported by parents in HICs, the major concerns were after-surgery costs, the cost of sibling childcare and life-long care of the sick child [25]. In our study, it was the cost of initial and subsequent surgery that was their greatest concern. About two-thirds of participating families were living below the WHO’s poverty line, and typically, one wage-earner was supporting a median of seven family members (range 4–16).

Our participants noted that patient and parent support groups could lead to a better understanding about, and support for, CHD management. The use of cost-effective strategies such as e-health may help to bypass geographical, travelling and financial barriers to maintain continuity of care, confirmed by other studies from Pakistan [26, 27].

There were important *institutional factors* identified, particularly, the lack of formal health-related education, and lack of a range of counselling services. Possibly due to lower health literacy, a poor understanding and awareness about CHD and related surgery was a key finding, and is consistent with other studies [28, 29]. Training health workers in early CHD screening and referral is viable in LMICs [30].

Other key omissions, also identified across international studies, include a lack of information about genetic counselling [29], unclear expectations about participation in sports [28], and unclear expectations about CHD surgery outcomes and potential for re-operation/related hospitalizations [31]. There is strong evidence of the positive role of anticipatory guidance in preparing patients and children, emotionally and practically, about what to expect along the CHD trajectory including diagnosis, treatment phases, and survivorship [32]. Providing clearer formal information channels, realistic expectations about living with a chronic cardiac condition and strategies for self-management may empower patients and parents leading to better coping and adaptation [33]. Structures and resources for emotional and psychological support are necessary as parents frequently feel vulnerable and powerless during their child’s illness [34].

Schools were raised as influential institutions that impacted the child’s experience of life after CHD surgery. Building a bank of e-resources or a point for liaison with schools could provide teachers with the confidence to advocate and plan for the particular needs of children/adolescents with CHD, in partnership with families.

*Sociocultural factors* were the most powerful and unique element to these findings. In our study, religion was key to perceptions about CHD and surgery, and a recent CHD study associated religion and spirituality with better HRQOL [35]. While parents from HICs have been reported to blame themselves for their child’s CHD [31], many of our participants perceived CHD as God’s will and considered themselves chosen by God for this unique experience. This is similar to reports on children with other congenital problems in Pakistan [36] and South Asian Muslim countries [37, 38].

While religion played a significant role in providing comfort to families, strictly-defined gender roles, gender discrimination and marriageability concerns were frequently raised. The attitudes towards CHD surgical scarring in our study findings were unique. Unlike a study from Canada where the majority of adolescents with CHD were comfortable disclosing their condition without the fear of being stigmatised [39], CHD is a source of stigma in Pakistan [40, 41]. Genetic diseases are negatively perceived [41], primarily due to decreased prospects for marriageability and in part due to a fear of genetic transmission to the next generation [40].

Higher levels of stigma towards people with chronic diseases in Pakistan are associated with lower levels of education and socioeconomic status [42]. Education and health literacy may therefore promote better understanding about chronic disease while providing direction for improved strategies for self-management and related sociocultural challenges [42]. Public health strategies on a mass scale could create awareness about the potential for genetic inheritance of congenital disease in a patrilineal society.

Pakistani society is highly gendered with women considered subordinate to men [43]. The patriarchal nature of Pakistani society had a significant influence on the experiences of parents with a female child affected by CHD and surgery, a unique finding not reported by qualitative studies elsewhere. Pakistan was rated 143 of 144 countries surveyed for gender equality in the 2017 World Economic Forum’s Global Gender Gap Report [44]. Discrimination towards women in Pakistan occurs across the life cycle [45], and reduces access to basic needs [46], and healthcare utilisation, with male children utilising twice as much healthcare as female children [47]. Mothers who have themselves experienced bias or neglect are more likely to have psychological morbidity, and are more likely to propagate a higher prioritisation of the needs of male children, while at the same time overprotecting girls [48].

Gender discrimination was also evident in concerns related to marriage. Marriage is considered a social obligation for women in Pakistan and provides social and economic protection. Females constitute only 22.4% of the workforce in Pakistan due to low literacy (45%). Priority is placed instead, on a women’s care of the family, making them financially dependent on the male family members, preferably their husband [43]. This is evident in our study where all the mothers were housewives. Unlike most HICs, there is no structured, social welfare system in Pakistan to provide support during times of vulnerability such as in illness or loss of employment [49]. Perhaps the stigma related to CHD and surgery [50] and the financial dependency of women on their husbands explain the considerable parental concerns about marriageability.

Constrained *policy and system resources* were influential in shaping participants’ experiences. Unlike most HICs, Pakistan does not have universal healthcare to deliver equitable healthcare access [51]. The limited public health services available are sub-standard. Eighty-six percent of people utilizing private health care pay either out of pocket or receive financial aid by private donors or philanthropic organizations, as confirmed by our participants. The private hospital setting provides financial support [partial or complete] to needy patients through zakat funds (Islamic ritual of donating money for the poor) and through individual CHD donations.

Despite the financial assistance received from private health services, the additional cost of travel to seek medical assistance adds to the financial burden for families in Pakistan, where more than 60% of the population resides in rural areas [52]. Parents described a lack of CHD services they believed impacted the current and future HRQOL of their child/adolescent arising from delayed CHD screening and surgery, along with poor access to reoperation and support for life-long care. This may help explain the lower emotional, physical and psychosocial functioning among a Pakistani child/adolescent cohort [6] compared with meta-analysis results from HICs.

Antenatal genetic technologies are increasingly available in LIMCs, however, the legal, ethical and social implications are not well-understood. In HICs there are practice guidelines and policies that support women as independent decision-makers on antenatal screening with a focus on self-determination and self-sufficiency. However, in Pakistan and other LMICs there is a relative policy vacuum, despite an acknowledged need for informed reproductive choice. Termination of pregnancy would be considered by couples in Pakistan, should a major in-utero defect be identified [53].

### Strengths and limitations

This study provides new insights into parent-identified factors that may influence decision-making about CHD surgery and the HRQOL for children/adolescents and in Pakistan. The following limitations are noted. The study participants belonged to a single, tertiary care private hospital. Though this facility provides care to patients across the country, some who received donor assistance, their experiences may differ from parents accessing public hospitals due to differences in socio-demographics. It is also acknowledged that many people with CHD in Pakistan may have no access to surgery.

Despite taking all precautions during interview transcription (in Urdu), translation (Urdu to English) and back translation (English to Urdu), there could be translation bias. In addition, the perceptions and issues described here represent parents’ descriptions only. Future research should be conducted in public settings, in rural areas and should report older children and adolescents’ perspectives.

## Conclusion

Intra- and interpersonal, institutional, sociocultural and policy factors had a pervasive and substantial impact in ways that could influence the HRQOL of children and adolescents following CHD surgery in the LMIC of Pakistan. Cost-effective strategies need to be developed to provide enhanced local community support to patients and their parents who are often distant from specialist paediatric centres. Clinicians should maintain their awareness of the particular challenges faced by girls needing CHD surgery so that the concerns of parents and children can be openly discussed. Elements of these findings may be transferrable to other resourced-constrained settings or to nations with similar cultural beliefs and attitudes.

## Supplementary information

**Additional file 1.** Semi structured interview guide for the parents.

**Additional file 2.** Consolidated criteria for reporting qualitative studies (COREQ): 32-item checklist.

**Additional file 3.** Partcipants charcteristics details.

## Data Availability

Data has been shared as supplementary files.
