# Supplementary material for: Exploring the influence of socio-cultural factors and environmental resources on the health related quality of life of children and adolescents after congenital heart disease surgery: parental perspectives from a low middle income country
Source: J Patient Rep Outcomes. 2020 Aug 28;4:72. doi: 10.1186/s41687-020-00239-0 (PMC7455647; doi:10.1186/s41687-020-00239-0)
Supplement: Supplementary file 1 — Additional file 1. Semi structured interview guide for the parents. [file 41687_2020_239_MOESM1_ESM.docx]

Supplementary file 1:

Semi structured interview guide for the parents:

1. In your view, what is quality of life for your child?

2. Can you talk about how you see your child’s congenital heart disease and surgery?

3. How does the community and society view your child’s congenital heart disease and surgery?

4. What is the most disturbing or bothersome aspect of your child’s surgery?

5. How do you/your child deal with these issues and problems?

6. What are some of the concerns that you see as very important for your child’s future years?

7. What do you think are the needs of patients with congenital heart disease managed with surgery

8. What would you like to suggest to the healthcare professionals to address those needs?
