# Supplementary material for: Exploring the influence of socio-cultural factors and environmental resources on the health related quality of life of children and adolescents after congenital heart disease surgery: parental perspectives from a low middle income country
Source: J Patient Rep Outcomes. 2020 Aug 28;4:72. doi: 10.1186/s41687-020-00239-0 (PMC7455647; doi:10.1186/s41687-020-00239-0)
Supplement: Supplementary file 2 — Additional file 2. Consolidated criteria for reporting qualitative studies (COREQ): 32-item checklist. [file 41687_2020_239_MOESM2_ESM.docx]

**Supplementary file 2**

Consolidated criteria for reporting qualitative studies (COREQ): 32-item checklist

| **No** | **Item** | **Guide questions/description** | **Comments** |
| --- | --- | --- | --- |
| **Domain 1: Research team and reflexivity** |  |  |  |
| Personal Characteristics |  |  |  |
| 1. | Interviewer/facilitator | Which author/s conducted the interview or focus group? | Laila Ladak conducted the interviews. |
| 2. | Credentials | What were the researcher's credentials? *E.g. PhD, MD* | **Laila Akbar Ladak**, PhD, MScN, BScN, RN  **Robyn Gallagher,** PhD, BA, MN, RN, FAHA, FESC  **Babar Sultan Hasan**, M.D., FAAP  **Khadija Awais,** MBBS  **Ahmed Abdullah,** MBBS  **Janice Gullick**, PhD, MArt, BFA, RN |
| 3. | Occupation | What was their occupation at the time of the study? | **Laila Akbar Ladak**,  PhD Candidate  Charles Perkins Centre, Sydney Nursing School, The University of Sydney, Australia  **Robyn Gallagher,** PhD, BA, MN, RN, FAHA, FESC  Professor of Nursing  Charles Perkins Centre, Sydney Nursing School, The University of Sydney, Australia  President-elect Australian Cardiovascular Health and Rehabilitation Association (ACRA)  Adjunct Professor, Faculty of Health, University of Technology, Sydney, Australia.  **Babar Sultan Hasan**,  Diplomate American Board of Pediatrics  Diplomate American Board of Pediatric Cardiology  Associate Professor  Department of Paediatrics and Child Health  The Aga Khan University, Pakistan  **Khadija Awais,**  Year 5 Medical Student  Medical College, The Aga Khan University, Pakistan  **Ahmed Abdullah,** Year 5 Medical Student  Medical College, The Aga Khan University, Pakistan  **Janice Gullick**,  Associate Professor, Coordinator, Master of Intensive Care Nursing.  Sydney Nursing School, The University of Sydney, Australia |
| 4. | Gender | Was the researcher male or female? | The research team comprised of 4 females and 2 males. However all the interviews were conducted by a female (LL). |
| 5. | Experience and training | What experience or training did the researcher have? | The primary investigator (LL) is a pediatric cardiac intensive care and a clinical trials research nurse who has extensive experience of both clinical and research. In addition, she is a PhD candidate at The University of Sydney. The supervision team for PhD have strong clinical, research and academia background in pediatric cardiology. |
| Relationship with participants |  |  | None of the team member had any relationship with the participants. |
| 6. | Relationship established | Was a relationship established prior to study commencement? | Since this paper is part of a large mixed methods study, a rapport was built with the participants during the quantitative data collection following which the eligible participants were approached and recruited for qualitative interviews. |
| 7. | Participant knowledge of the interviewer | What did the participants know about the researcher? e*.g. personal goals, reasons for doing the research* | Participants were sent the study’s invitation letter (details about the study) with a follow up call to provide an understanding about the study objectives and outcomes. |
| 8. | Interviewer characteristics | What characteristics were reported about the interviewer/facilitator? e.g. *Bias, assumptions, reasons and interests in the research topic* | The following statement has been added under “Strength and limitations” section:  “Despite taking all precautions during interview transcription (in Urdu), translation (Urdu to English) and back translation (English to Urdu), there could be translation bias.”  In addition, the following text has been mentioned under the “Rigour” section to highlight the measures taken to enhance the rigour of the study and maintain the overall data management:  “A content analysis checklist ([Elo et al. 2014](#_ENREF_12)) was used to improve trustworthiness and to guide the preparation, organisation and reporting of the data with additional guidance from the COREQ (COnsolidated criteria for REporting Qualitative research) ([Tong, Sainsbury, and Craig 2007](#_ENREF_44)). Credibility is supported by maximum variation sampling for age, gender and CHD type and inclusion of verbatim exemplars. Dependability was enhanced by interviews collected within a confined period to reduce design-induced changes. Conformability was supported by initial independent coding by two researchers (LL & JG), later rechecking by another researcher (RG) and by the back translation of key exemplars to confirm accuracy (BH). Transferability is facilitated by a clear description of participant characteristics and a deliberate focus and description of this LMIC setting. |
| **Domain 2: study design** |  |  |  |
| Theoretical framework |  |  | Social Ecological Model was used to understand the influence of individual, sociocultural and environmental factors that influence health-related decision-making and the HRQOL for children or adolescents after CHD surgery in Pakistan. |
| 9. | Methodological orientation and Theory | What methodological orientation was stated to underpin the study? *e.g. grounded theory, discourse analysis, ethnography, phenomenology, content analysis* | It was a qualitative study and content analysis was conducted for the analysis. |
| Participant selection |  |  |  |
| 10. | Sampling | How were participants selected? *e.g. purposive, convenience, consecutive, snowball* | Participants were parents of CHD patients aged 2-18 years who were able to understand English or Urdu and were willing to travel to the study setting for a face-to-face interview. This nested sample, a subset of parents from the larger study, were recruited using a stratified, purposive strategy to achieve diversity in CHD diagnoses (simple, moderate and complex CHD) and child age (children and adolescents). Parents from twenty-seven families (both mother and father where possible) were approached. Seven pairs of parents declined to participate due to lack of time. Of the remaining families, data saturation achieved with the 18th interview. Two additional interviews were completed, increasing participation to 20 families, but there were no further novel findings in these subsequent interviews. The 20 families included 18 parent dyads (mother and father) and two single mothers, to reach a total of 38 participants. |
| 11. | Method of approach | How were participants approached? e*.g. face-to-face, telephone, mail, email* | A face to face semi-structured interviews were conducted. |
| 12. | Sample size | How many participants were in the study? | Parents from twenty-seven families (both mother and father where possible) were approached. Seven pairs of parents declined to participate due to lack of time. Of the remaining families, data saturation achieved with the 18th interview. Two additional interviews were completed, increasing participation to 20 families, but there were no further novel findings in these subsequent interviews. The 20 families included 18 parent dyads (mother and father) and two single mothers, to reach a total of 38 participants. |
| 13. | Non-participation | How many people refused to participate or dropped out? Reasons? | Parents from twenty-seven families (both mother and father where possible) were approached. Seven pairs of parents declined to participate due to lack of time. |
| Setting |  |  |  |
| 14. | Setting of data collection | Where was the data collected? e*.g. home, clinic, workplace* | Parents were interviewed together in a private room to facilitate free expression and to maintain confidentiality. |
| 15. | Presence of non-participants | Was anyone else present besides the participants and researchers? | Nobody except the participants and the researcher were present during the interviews. |
| 16. | Description of sample | What are the important characteristics of the sample? *e.g. demographic data, date* | The sample characteristics includes mean age, education, income, family structure and occupation (highlighted in table 1 and written text in results section). In addition, a supplementary file is also provided for details about the clinical and sociodemographic data of the CHD surgical patients. |
| Data collection |  |  |  |
| 17. | Interview guide | Were questions, prompts, guides provided by the authors? Was it pilot tested? | Yes, a semi-structured interview guide was used to explore the impact of the socio-cultural and resource environment and surroundings on participants’ understandings and perceptions of their child’s HRQOL, experiences following surgery and the issues they and their children faced arising from CHD and surgery. To ensure for the interview guide’s face and content validity, it was piloted on four ineligible parents. There was one question which was revised for better clarity and ease to comprehend for the parents. |
| 18. | Repeat interviews | Were repeat interviews carried out? If yes, how many? | None of the interviews were repeated. |
| 19. | Audio/visual recording | Did the research use audio or visual recording to collect the data? | Yes, the interviews were audio recorded. |
| 20. | Field notes | Were field notes made during and/or after the interview or focus group? | Yes, the field notes were maintained during the interviews. |
| 21. | Duration | What was the duration of the interviews or focus group? | Mean interview time was 50 minutes (range 30 to 60 minutes). |
| 22. | Data saturation | Was data saturation discussed? | Yes, data saturation is highlighted in the manuscript under “Setting and participants” section:  “Participants were parents of CHD patients aged 2-18 years who were able to understand English or Urdu and were willing to travel to the study setting for a face-to-face interview. This nested sample, a subset of parents from the larger study, were recruited using a stratified, purposive strategy to achieve diversity in CHD diagnoses (simple, moderate and complex CHD) and child age (children and adolescents). Parents from twenty-seven families (both mother and father where possible) were approached. Seven pairs of parents declined to participate due to lack of time. Of the remaining families, data saturation achieved with the 18th interview. Two additional interviews were completed, increasing participation to 20 families, but there were no further novel findings in these subsequent interviews. The 20 families included 18 parent dyads (mother and father) and two single mothers, to reach a total of 38 participants.” |
| 23. | Transcripts returned | Were transcripts returned to participants for comment and/or correction? | No, this was not required. |
| **Domain 3: analysis and findings** |  |  |  |
| Data analysis |  |  |  |
| 24. | Number of data coders | How many data coders coded the data? | Conformability was supported by initial independent coding by two researchers (LL & JG), later rechecking by another researcher (RG) and by the back translation of key exemplars to confirm accuracy. |
| 25. | Description of the coding tree | Did authors provide a description of the coding tree? | Yes, description of the coding tree is mentioned under the “Results” section and has also been illustrated diagrammatically in figure 1. |
| 26. | Derivation of themes | Were themes identified in advance or derived from the data? | Themes were identified from the data. Figure 1 also provides a diagrammatic illustration of the identified themes and sub themes. |
| 27. | Software | What software, if applicable, was used to manage the data? | Data management was done manually and no software was used. |
| 28. | Participant checking | Did participants provide feedback on the findings? | No |
| Reporting |  |  |  |
| 29. | Quotations presented | Were participant quotations presented to illustrate the themes / findings? Was each quotation identified? e*.g. participant number* | Yes, participant quotations were presented to support the identified themes and sub themes. In addition, the participants are referred to by pseudonym. |
| 30. | Data and findings consistent | Was there consistency between the data presented and the findings? | Yes, there was consistency between the data presented and the findings. |
| 31. | Clarity of major themes | Were major themes clearly presented in the findings? | Yes, major themes have been clearly presented in the findings under “Results” section and has also been illustrated diagrammatically in figure 1. |
| 32. | Clarity of minor themes | Is there a description of diverse cases or discussion of minor themes? | Yes, sub themes and codes have also been highlighted. |
